# Sophorae Flos extract inhibits RANKL-induced osteoclast differentiation by suppressing the NF-κB/NFATc1 pathway in mouse bone marrow cells

**DOI:** 10.1186/s12906-016-1550-x

**Published:** 2017-03-23

**Authors:** Jeong-Mi Kim, Jung-Han Lee, Guem-San Lee, Eun-mi Noh, Hyun-Kyung Song, Dong Ryun Gu, Seong-Cheol Kim, Seoung Hoon Lee, Kang-Beom Kwon, Young-Rae Lee

**Affiliations:** 1 Center for Metabolic Function Regulation (CMFR), Wonkwang University School of Medicine, Iksan, 570-749 Republic of Korea; 2 Department of Rehabilitation Medicine of Korean Medicine, Wonkwang University School of Korean Medicine, Iksan City, Jeonbuk 570-749 Republic of Korea; 3 Department of Herbology, Wonkwang University School of Korean Medicine, Iksan, 570-749 Republic of Korea; 4 Department of Oral Microbiology and Immunology, College of Dentistry, Wonkwang University, Iksan, 570-749 Republic of Korea; 5 Department of Korean Physiology, Wonkwang University School of Korean Medicine, Iksan City, Jeonbuk 570-749 Republic of Korea; 6 Department of Oral Biochemistry, and Institute of Biomaterial-Implant, College of Dentistry, Wonkwang University, Iksan, 570-749 Republic of Korea; 7 Integrated Omics institute, Wonkwang University, Iksan, 570-749 Republic of Korea

**Keywords:** Sophorae Flos (SF), Osteoclast, NF-κB, NFATc1, PLCγ2

## Abstract

**Background:**

Sophorae Flos (SF) is a composite of flowers and buds of *Styphnolobium japonicum* (L.) Schott and has been used in traditional Korean and Chinese medicine for the treatment of hemostasis and inflammation. Previous studies reported that SF possesses anti-obesity properties, as well as anti-allergic, anti-proliferative, and anti-inflammatory activities. However, the effect of SF in bone resorption has not been studies. In this study, we examined the potential of SF extract (SFE) to inhibit receptor activator of NF-κB ligand (RANKL) -induced osteoclast differentiation in cultured mouse-derived bone marrow macrophages (BMMs).

**Methods:**

BMMs, that act as osteoclast precursors, were cultured with M-CSF (50 ng/ml) and RANKL (100 ng/ml) for 4 days to generate osteoclasts. Osteoclast differentiation was measured by tartrate-resistant acidic phosphatase (TRAP) staining and the TRAP solution assay. Osteoclast differentiation marker genes were analyzed by the quantitative real-time polymerase chain reaction analysis. RANKLs signaling pathways were confirmed through western blotting.

**Results:**

SFE significantly decreased osteoclast differentiation in a dose-dependent manner. SFE inhibited RANKL-induced osteoclastogenesis by suppressing NF-κB activation. By contrast, SFE did not affect phospholipase C gamma 2 or subsequent cAMP response element binding activation. SFE inhibited the RANKL-induced expression of nuclear factor of activated T cells c1 (NFATc1).

**Conclusions:**

SFE attenuated the RANKL-mediated induction of NF-κB through inhibition of IκBα phosphorylation, which contributed to inhibiting of RANKL-induced osteoclast differentiation through downregulation of NFATc1.

## Background

Osteoclasts differentiate from monocyte/macrophage lineage hematopoietic precursor cells at various stages including proliferation, migration, fusion, and activation [1]. Osteoclasts are specialized as the only bone-resorbing cell type and increased numbers are implicated in the development of bone loss-accompanied diseases such as osteoporosis, periodontitis, rheumatoid arthritis, osteosarcoma, and bone cancer metastases [2–4]. Pharmaceutical inhibition of osteoclast differentiation is a current therapeutic approach for the treatment of bone loss-related diseases.

Macrophage colony-stimulating factor (M-CSF) and receptor activator of NF-κB ligand (RANKL) are two cytokines secreted mainly by osteoclast. Both are differentiation factors [5, 6]. RANKL binds specifically to receptor activator of nuclear factor (NF)-κB (RANK) that mediates osteoclastogenesis by subsequent signal transduction to intracellular molecules through the TRAF6 adaptor protein. Thereafter, the RANKL/RANK interaction activates extracellular signal-regulated kinase (ERK), p38, c-Jun N-terminal kinase (JNK), Akt, and NF-κB [7–9]. Ultimately, these signal transduction pathways lead to the expression and activation of transcription factors such as nuclear factor of activated T cells c1 (NFATc1) and activator protein-1 (AP-1), both of which are involved in the expression of genes specific to osteoclasts [9–11].

Sophorae Flos (SF), the dried flower buds of *Styphnolobium japonicum* (L.) Schott, is a well-known herb in traditional Chinese medicine. It has been used in the treatment of bleeding-related disorders such as hematochezia, hemorrhoidal bleeding, dysfunctional uterine bleeding, and diarrhea [12]. Several phytochemical investigations have revealed that natural products from *S. japonicum* (L.) Schott fruit extracts contain various flavonoids, including sophoricoside, genistin, genistein, kaempferol, rutin, and quercetin [13, 14]. In both pharmacological studies and clinical practice, *S. japonicum* (L.) Schott exhibits anti-tumor, anti-inflammatory, anti-platelet, and anti-obesity activities [15–18].

Previous studies indicate that pro-inflammatory cytokines, including IL-17, TNF-*α*, IL-1, IL-4, and IFN-*γ*, that are induced during T-cell-mediated immune responses, directly control the expression of RANKL on osteoblasts and that inflammation affects bone metabolism [1, 19, 20]. Although *S. japonicum* (L.) Schott has anti-inflammatory activity, effect on bone metabolism has been studie infrequently. The exceptions are studies showing that dichloromethane extracts of Sophora japonica L. stimulate osteoblast differentiation in mesenchymal stem cells [21]. In addition, recent studies show that such extracts prevent bone loss, partly by inhibiting osteoclastic activity [21, 22]. However, the potential anti-osteoclast differentiation mechanisms of SF have not been defined clearly.

In our study, we confirmed the inhibitory effects of SF extract (SFE) on RANKL-mediated osteoclast differentiation, provided molecular mechanisms for its activity, and suggested possibilities for the use of SF as a traditional medicine against bone disorders, such as osteoporosis, RA, and periodontitis.

## Methods

### Experimental animals

BALB/c mice (Orient Bio, SeungNam, Korea) were used for all experiments, including osteoclast generation. All mouse studies were performed using protocols approved by the Animal Care and Use Committee of Wonkwang University.

### Reagents

Recombinant murine sRANK Ligand and M-CSF were purchased from PeproTech (Rocky Hill, NJ, USA). Fetal bovine serum, α-minimal essential medium, and supplements were obtained from Gibco (Rockford, IL, USA).

### Preparation of SFEs

Flower buds of *S. japonicum* (L.) Schott were purchased from Kwangmyungdang Medicinal Herbs (Ulsan, Korea) and authenticated by Prof. G.S. Lee. SF was extracted from 50 g of *S. japonicum* flower buds using the reflux method with ethanol. The extract was evaporated and then freeze-dried. The yield of the final extract was 2.56% (w/w).

### Cell viability assay

In 96-well plates, bone marrow-derived macrophages (BMMs) were treated with different concentrations of SFE (0, 25, 50, 100, and 200 μg/ml) for 1 day, or with 100 μg/ml SFE under M-CSF treatment for 4 days. Next, cells were then incubated with EZ-Cytox reagent (Itsbio, Korea) for 4 h at 37 °C. After incubation, cell viability was measured using a microplate reader (Sunrise™, Tecan, Switzerland) at 450 nm.

### *In vitro* osteoclast differentiation

Collected from mice tibiae and femur, BMMs were cultured with M-CSF (30 ng/ml). After 3 days, attached BMMs were used as osteoclast precursor. To form osteoclasts, BMMs were treated with M-CSF (50 ng/ml) and RANKL (100 ng/ml) and cultured for 4 days [23]. For TRAP staining, cells were fixed with 10% formalin and stained. Total TRAP activity was measured at an absorbance of 405 nm using p-nitrophenyl phosphate (Sigma Aldrich, St. Louis, MO, USA) as a substrate.

### Real-time quantitative polymerase chain reaction (qRT-PCR)

Total RNA was isolated from cells using the Trizol reagent (Invitrogen, Carlsbad, CA, USA). One microgram of total RNA was synthesized to first strand cDNA using a PrimeScript™ RT reagent kit (TaKaRa Bio, Shiga, Japan). qRT-PCR was performed using the SYBR Green and the StepOnePlus Real-Time PCR System (Applied Biosystems, Foster City, CA, USA). All results were normalized to the housekeeping gene glyceraldehyde 3-phosphate dehydrogenase (*Gapdh)*. PCR primers used were: mouse *Acp5* (sense: 5′-CTG GAG TGC ACG ATG CCA GCG ACA-3′ and antisense: 5′-TCC GTG CTC GGC GAT GGA CCA GA-3′); *Oscar* (sense: 5′-GGG GTA ACG GAT CAG CTC CCC AGA-3′ and antisense: 5′-CCA AGG AGC CAG AAC GTC GAA ACT-3′); *CtsK* (sense: 5′-ACG GAG GCA TTG ACT CTG AAG ATG-3′ and antisense: 5′-GTT GTT CTT ATT CCG AGC CAA GAG-3′); *Tm7sf4* (sense: 5′-TGG AAG TTC ACT TGA AAC TAC GTG-3′ and antisense: 5′-CTC GGT TTC CCG TCA GCC TCT CTC-3′); *Atp6v0d2* (sense: 5′-TCA GAT CTC TTC AAG GCT GTG CTG-3′ and antisense: 5′-GTG CCA AAT GAG TTC AGA GTG ATG-3′); *Nfatc1* (sense: 5′-CTC GAA AGA CAG CAC TGG AGC AT-3′ and antisense: 5′-CGG CTG CCT TCC GTC TCA TAG-3′); and *Gapdh* (sense: 5′-TGC CAG CCT CGT CCC GTA GAC-3′ and antisense: 5′-CCT CAC CCC ATT TGA TGT TAG-3′).

### Western blot analysis

Cells were lysed with RIPA Lysis buffer (Pierce Biotechnology, Rockford, IL, USA). The protein concentration in the supernatants was determined using the Bradford method [24]. Protein samples (30 μg) were separated in sodium dodecyl sulfate-polyacrylamide gels and transferred to polyvinylidene fluoride membranes (GE, Buckinghamshire, UK) using a western blot apparatus. Each membrane was blocked in blocking buffer (2% bovine serum albumin or 5% skim milk) and then incubated with primary antibody ( phospholipase C gamma 2 (PLCγ2), p-ERK, p-JNK, p-p38, cAMP response element binding (CREB), p-IκBα, p-PLCγ2, ERK, JNK, p38, CREB (Cell signaling Technology, Danvers, MA, USA), NFATc1, c-fos (Santa Cruz Biotechnology, Santa Cruz, CA, USA), β-actin (Sigma-Aldrich)). Horseradish peroxidase-conjugated IgG (1:2000 dilutions) was used as the secondary antibody. Immunoreactivity was detected using a Mini HD6 image analyzer (Uvitec Cambridge, UK).

### Statistical analysis

Results were analyzed using Student’s two-tailed t-test. Data are presented as means ± standard deviation (SD). *P*-values less than 0.05 were considered significant.

## Results

### Cytotoxic effects of SFE

**Fig. 1:**
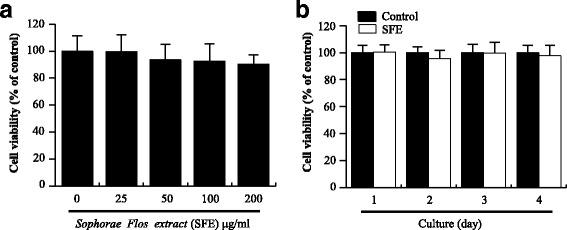
Effects of Sophorae Flos extract (SFE) on bone marrow marcrophages. **a** BMMs were cultured with various concentrations of SFE for 1 day. **b** BMMs were cultured with or without (control) 100 μg/ml SFE for 4 days. Cell viability was measured as described in methods. Data from three independent experiments are expressed as relative proliferation (% of control) ± SD

To investigate the cytotoxicity of SFE on BMMs (osteoclast precursor), it treated with several concentrations of SFE (0, 25, 50, 100, and 200 μg/ml) for 1 day. Concentrations of SFE, up to 100 μg/ml, did not cause any significant change in cell viability (Fig. 1a). BMMs were also treated with 100 μg/ml SFE and cell viability was measured daily for 4 days. There were no significant differences in viability between control and SFE at any day (Fig. 1b).

### SFE treatment suppressed RANKL-mediated osteoclastogenesis in a dose-dependent manner

**Fig. 2:**
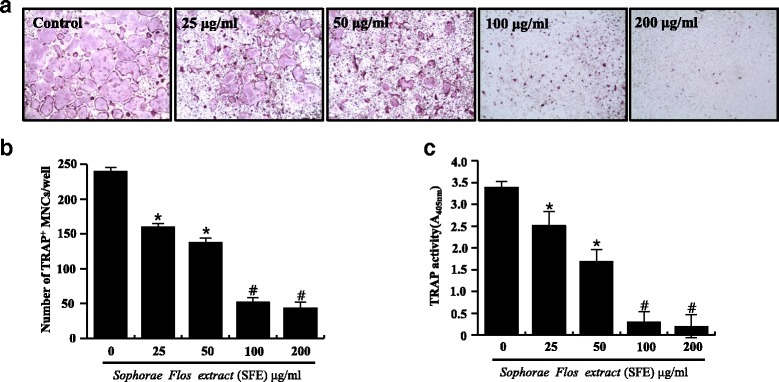
Effects of Sophorae Flos extract (SFE) on osteoclast differentiation. Bone marrow macrophages were cultured with various concentrations of SFE and treated with RANKL (100 μg/ml) and M-CSF (50 μg/ml) for 4 days. **a** Osteoclasts stained for tartrate-resistant acidic phosphatase (TRAP). **b** TRAP^+^ multinuclear cells (MNCs) with more than three nuclei were considered to be mature osteoclasts. **c** Total TRAP activity from TRAP^+^ mono-, di-, and multinuclear cells was quantified as described in methods. Data from three independent experiments are expressed as mean ± SD. *P < 0.05, ^#^ *P* < 0.01 versus control (0 μg/ml SFE)

To investigate the effects of SFE on osteoclast differentiation, RANKL-stimulated BMMs were treated with SFE at the indicated concentrations for 4 days (Fig. 2a). After incubation, osteoclast differentiation and formation were measured by TRAP staining and the total TRAP activity assay. TRAP^+^ multimuclear cells present in each well were identified by the presence of more than three nuclei and a cell size larger than 100 μm in diameter, and were counted. Total TRAP activity was measured in fused and non-fused cells. SFE treatment markedly inhibited RANKL-induced osteoclast formation from BMMs in a dose-dependent manner (Fig. 2a, b). Total TRAP activity of osteoclasts was reduced significantly by SFE in a concentration dependent manner (Fig. 2c). These results suggest that SFE is able to repress osteoclast differentiation.

### Abrogation of differentiation-related gene expression by SFE

**Fig. 3:**
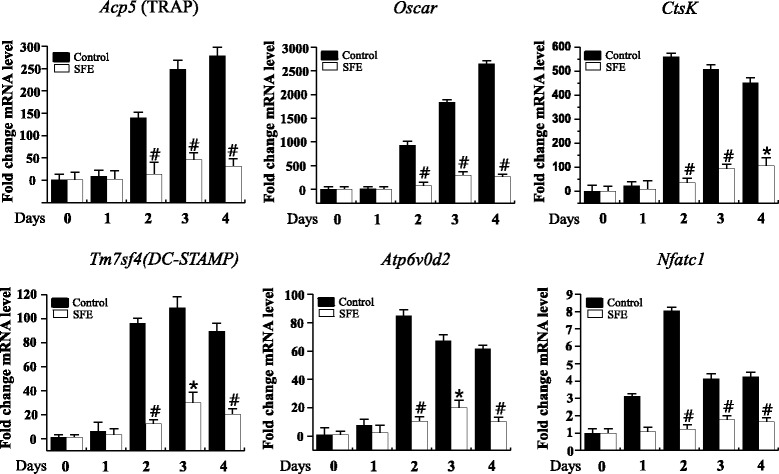
Effects of Sophorae Flos extract (SFE) on the expression of osteoclast differentiation marker genes. Bone marrow macrophages were cultured with RANKL and M-CSF treatment in the presence or absence of SFE (100 μg/ml) for 4 days. The expression of marker genes of osteoclast differentiation was measured by real-time quantitative PCR. Target gene mRNA levels were normalized to GAPDH and are presented as fold change from control (0 μg/ml SFE). Data are expressed as mean ± SD and are representative of at least three independent experiments. *P < 0.05, #*P* < 0.01 versus control

*Acp5* (TRAP), *Oscar*, *Ctsk*, *Tm7sf4* (dendritic cell-specific transmembrane protein, DC-STAMP), and *Atp6v0d2* are well-known as osteoclast differentiation-related marker genes crucial for cell motility, fusion, and bone resorptive activities. To evaluate the inhibitory effect of SFE on RANKL-induced osteoclast differentiation, the expression of these marker genes and a major transcription factor *Nfatc1,* was measured during RANKL-induced osteoclast differentiation. SFE dramatically inhibited the expression of all tested osteoclast differentiation-related marker genes, as well as *Nfatc1* (Fig. 3). These results suggest that SFE affects the differentiation-mediating signal pathway from an early-stage of osteoclastogenesis.

### Suppression of NFATc1 expression by SFE-mediated NF-κB inactivation

**Fig. 4:**
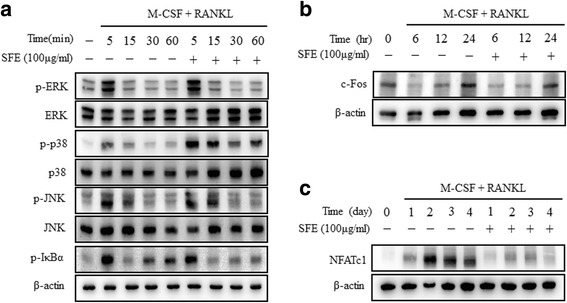
Effects of Sophorae Flos extract (SFE) on RANKL-induced intracellular signaling and expression of c-fos and NFATc1 in osteoclasts. Bone marrow macrophages were cultured with RANKL and MCSF in the presence or absence of SFE (100 μg/ml) for 4 days. Protein expression levels were evaluated by western blot analysis. **a** Activation of MAPKs and NF-κB measured using by their respective antibodies. Expression of (**b**) c-fos and (**c**) NFATc1 detected by the respective antibodies. All data are representative of at least three independent experiments

**Fig. 5:**
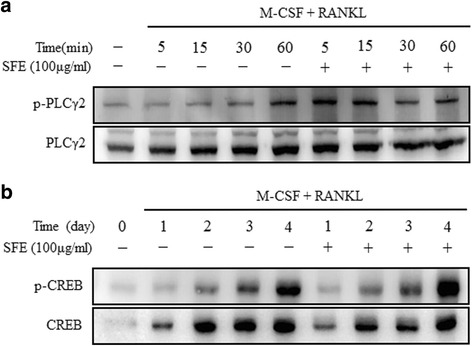
Sophorae Flos extract (SFE) does not inhibit RANKL-mediated activation of PLCγ2 and CREB in osteoclasts. Bone marrow macrophages were cultured with M-CSF and RANKL in presence or absence of SFE (100 μg/ml) for 4 days. Lysate (30 μg protein) was subjected to SDS-PAGE and the phosphorylation of (**a**) PLCγ2 and (**b**) CREB analyzed by western blotting. All data are representative of at least three independent experiments

Osteoclast differentiation is regulated by the recruitment of multiple downstream signaling molecules, including PLCγ2, p38, JNK, and ERK, as well as the transcription factors NF-κB, NFATc1, and c-fos, in response to RANKL and its binding to RANK [25]. We confirmed the effects of SFE on RANKL-induced early signaling pathways. First, we confirmed the activation of mitogen-activated protein kinases (MAPKs) and NF-κB by western blotting (Fig. 4). SFE showed had no effect on MAPK activation. By contrast, IκBα phosphorylation, proportional to the NF-κB pathway activity, was diminished in SFE-treated compared with control cells (Fig. 4a). Next, we investigated the expression levels of NFATc1 and c-fos proteins. In SFE-treated cells, the expression of c-fos protein was unchanged during RANKL-induced osteoclast differentiation (Fig. 4b). However, the expression of NFATc1 protein was inhibited dramatically by SFE (Fig. 4c). Concomitantly, we found that SFE failed to inhibit the activation of PLCγ2 and CREB during RANKL-induced osteoclast differentiation (Fig. 5a, b). These results suggest that SFE affects the regulation of RANKL-induced osteoclast differentiation through the NF-κB pathway.

## Discussion

SF has been used commonly in traditional medicine because of its various hemostatic, anti-inflammatory, and anti-oxidative effects [26]. Additionally, recent studies indicated that *S. japonicum* (L.) Schott extracts showed preventive effects against bone loss, partly by inhibiting osteoclastic activity [19, 20]. However, the inhibitory potential and molecular mechanisms of SF on RANKL-induced osteoclast differentiation have not been elucidated. Here, we demonstrated that the inhibitory effect of SFE was elicited through a reduction of NFATc1 expression during the differentiation of osteoclasts, cells responsible for bone destruction and associated with inflammation-related bone diseases.

BMMs are precursor cells that differentiate into osteoclasts in response to RANKL, which is expressed in osteoblasts, osteocytes, and T cells, and a critical factor in osteoclastogenesis [27].

The RANKL/RANK interaction recruits multiple intracellular signaling molecules that regulate osteoclast differentiation, including MAPK, NF-κB, AP-1, TRAFs, NFATc1, and ionized calcium, with NF-κB being the most important factor [1, 28].

NF-κB is transcription factor and an inducible dimeric protein consisting of two subunits, p65 and p50 [29]. In unstimulated cells, NF-κB is located in the cytoplasm in a dormant form complexed with its inhibitory factor, IκB. Various inducers can dissociate this complex, presumably by phosphorylating IκB, resulting in the release of NF-κB. NF-κB then translocates into the nucleus, where it binds to specific DNA sites to regulate gene transcription. In several studies, NF-κB was shown to play a key role in osteoclastogenesis [30, 31], and its suppression affected NFATc1 expression. Takatsuna et al. have shown that the NF-κB inhibitor, (-)-dehydroxymethylepoxyquinomicin regulates RANKL-induced osteoclastogenesis through downregulation of NFATc1 [32]. In the current study, we found that IκBα phosphorylation was inhibited by SFE, whereas the induction of MAPK was unaffected (Fig. 4).

Multiple previous studies have established that NFATc1 is a master executor of RANKL-mediated osteoclast differentiation and activation. Stimulation of BMM s by RANKL increases the expression of NFATc1 through c-Fos and auto amplification [11, 23, 33–35]. NFATc1 also gradually induces the expression of osteoclast-specific genes, including *Acp5* (encoding TRAP), *Oscar*, *Tm7sf4* (encoding DC-STAMP), *Atp6v0d2*, and *Ctsk* [11, 33, 34, 36]. The present data suggest that SFE suppressed the induction of *Acp5*, *Oscar*, *Tm7sf4*, *Atp6v0d2*, and *Ctsk* (Fig. 3). In addition, SFE suppressed the RANKL-mediated induction of NFATc1 mRNA and protein expression, although the expression of c-fos was not unaffected (Figs. 3 and 4).

RANKL/RANK binding activates PLC*γ*2 and induces calcium ion signaling, followed by CREB phosphorylation [23, 25, 37]. CREB is crucial factor for the RANKL-stimulated induction of NFATc1 and c-Fos in osteoclast precursors [37]. Nevertheless, in our study, SFE failed to inhibit PLCγ2 and CREB activation, which are essential signaling molecules for osteoclast differentiation, through the repression of c-Fos and NFATc1 expression. These results indicate that SFE is an effective inhibitory agent of osteoclast differentiation through its control of NF-κB induction following RANKL/RANK binding.

## Conclusions

Our results demonstrate that SFE reduces the RANKL-mediated induction of the NF-κB pathway by inhibiting of IκBα phosphorylation. This effect, in turn, contributes to the downregulation of NFATc1 and inhibition of RANKL-induced osteoclast differentiation. These findings reveal SFE as an effective traditional therapeutic medicine for the treatment of inflammatory bone diseases, such as osteoporosis, rheumatoid arthritis, and periodontitis.

## Abbreviations

AP-1: Activator protein-1
BMMs: Bone marrow macrophages
BMs: Bone marrow cells
DC-STAMP: Dendritic cell-specific transmembrane protein
ERK: Extracellular signal-regulated kinase
FBS: Fetal bovine serum
JNK: c-Jun N-terminal kinase
M-CSF: Macrophage colony-stimulating factor
NFATc1: Nuclear factor of activated T cells c1
PBS: Phosphate-buffered saline
PLCγ2: Phospholipase C gamma 2
RA: Rheumatoid arthritis
RANK: Receptor activator of NF-κB
RANKL: RANK ligand
SD: Standard deviation
SF: Sophorae flos
SFE: SF extract
TRAP^+^ MNCs: TRAP positive-multinuclear cells
α-MEM: α-minimal essential medium
