# Erratum to: Sophorae Flos extract inhibits RANKL-induced osteoclast differentiation by suppressing the NF-κB/NFATc1 pathway in mouse bone marrow cells

**DOI:** 10.1186/s12906-017-1745-9

**Published:** 2017-04-28

**Authors:** Jeong-Mi Kim, Jung-Han Lee, Guem-San Lee, Eun-mi Noh, Hyun-Kyung Song, Dong Ryun Gu, Seong-Cheol Kim, Seoung Hoon Lee, Kang-Beom Kwon, Young-Rae Lee

**Affiliations:** 1 Center for Metabolic Function Regulation (CMFR), Wonkwang University School of Medicine, Iksan, 570, –749 Republic of Korea; 2 Department of Rehabilitation Medicine of Korean Medicine, Wonkwang University School of Korean Medicine, Iksan City, Jeonbuk 570-749 Republic of Korea; 3 Department of Herbology, Wonkwang University School of Korean Medicine, Iksan, 570-749 Republic of Korea; 4 Department of Oral Microbiology and Immunology, College of Dentistry, Wonkwang University, Iksan, 570-749 Republic of Korea; 5 Department of Korean Physiology, Wonkwang University School of Korean Medicine, Iksan City, Jeonbuk 570-749 Republic of Korea; 6 Department of Oral Biochemistry, and Institute of Biomaterial-Implant, College of Dentistry, Wonkwang University, Iksan, 570-749 Republic of Korea; 7 Integrated Omics institute, Wonkwang University, Iksan, 570-749 Republic of Korea

## Erratum

Upon publication of the original article [1], it was noticed that the following statement was included “The present study was supported by Wonkwang University in 2014.” The year given should read 2015. This has since been corrected in this erratum.
